# From intensive care to step-down units: Managing patients throughput in response to COVID-19

**DOI:** 10.1093/intqhc/mzaa091

**Published:** 2020-08-11

**Authors:** Vanni AGNOLETTI, Emanuele RUSSO, Alessandro CIRCELLI, Marco BENNI, Giuliano BOLONDI, Costanza MARTINO, Domenico P SANTONASTASO, Etrusca BROGI, Beniamino PRATICÒ, Federico COCCOLINI, Paola FUGAZZOLA, Luca ANSALONI, Emiliano GAMBERINI

**Affiliations:** Department of Anesthesia and Intensive Care, M Bufalini Hospital, Viale Ghirotti 285, 47521, Cesena, Italy; Department of Anesthesia and Intensive Care, M Bufalini Hospital, Viale Ghirotti 285, 47521, Cesena, Italy; Department of Anesthesia and Intensive Care, M Bufalini Hospital, Viale Ghirotti 285, 47521, Cesena, Italy; Department of Anesthesia and Intensive Care, M Bufalini Hospital, Viale Ghirotti 285, 47521, Cesena, Italy; Department of Anesthesia and Intensive Care, M Bufalini Hospital, Viale Ghirotti 285, 47521, Cesena, Italy; Department of Anesthesia and Intensive Care, M Bufalini Hospital, Viale Ghirotti 285, 47521, Cesena, Italy; Department of Anesthesia and Intensive Care, M Bufalini Hospital, Viale Ghirotti 285, 47521, Cesena, Italy; Department of Anesthesia and Intensive Care, University of Pisa, Via Piero Trivella, 56124, Pisa, Italy; Department of Internal Medicine, M Bufalini Hospital, Viale Ghirotti 285, 47521, Cesena, Italy; Department of Surgery, University of Pisa, Via Piero Trivella, 56124, Pisa, Italy, and; General, Emergency and Trauma Department, M Bufalini Hospital, Viale Ghirotti 285, 47521, Cesena, Italy; General, Emergency and Trauma Department, M Bufalini Hospital, Viale Ghirotti 285, 47521, Cesena, Italy; Department of Anesthesia and Intensive Care, M Bufalini Hospital, Viale Ghirotti 285, 47521, Cesena, Italy

**Keywords:** coronavirus, intensive care units, step-down units, length of stay, hospital bed capacity, mass casualty incidents

## Abstract

**Quality problem or issue:**

The on-going COVID-19 pandemic may cause the collapse of healthcare systems because of unprecedented hospitalization rates.

**Initial assessment:**

A total of 8.2 individuals per 1000 inhabitants have been diagnosed with COVID-19 in our province. The hospital predisposed 110 beds for COVID-19 patients: on the day of the local peak, 90% of them were occupied and intensive care unit (ICU) faced unprecedented admission rates, fearing system collapse.

**Choice of solution:**

Instead of increasing the number of ICU beds, the creation of a step-down unit (SDU) close to the ICU was preferred: the aim was to safely improve the transfer of patients and to relieve ICU from the risk of overload.

**Implementation:**

A nine-bed SDU was created next to the ICU, led by intensivists and ICU nurses, with adequate personal protective equipment, monitoring systems and ventilators for respiratory support when needed. A second six-bed SDU was also created.

**Evaluation:**

Patients were clinically comparable to those of most reports from Western Countries now available in the literature. ICU never needed supernumerary beds, no patient died in the SDU, and there was no waiting time for ICU admission of critical patients. SDU has been affordable from human resources, safety and economic points of view.

**Lessons learned:**

COVID-19 is like an enduring mass casualty incident. Solutions tailored on local epidemiology and available resources should be implemented to preserve the efficiency and adaptability of our institutions and provide the adequate sanitary response.

## Quality problem

There are fears that the on-going coronavirus disease (COVID-19) pandemic may cause the collapse of healthcare systems around the world. Hospitals have had to apply emergency plans to implement the available resources, including the increase in the capacity of intensive care units (ICUs).

## Initial assessment

The Italian National Healthcare Service (NHS) relies on having 8.42 ICU beds for every 100 000 inhabitants [1]. This is the report of a public hospital with a capacity of 450 beds, 18 of which are in the ICU. It acts as a level 1 trauma centre and a hub for stroke and neurosurgery. It serves an area of 1.2 million inhabitants and is located just 35 km north-west of one of the main spots of COVID-19 contagion in Italy.

A total of 784 individuals have been diagnosed with COVID-19 (nasopharyngeal swabs) in our Province until the 20th of June (8.2 cases per 1000 inhabitants). The hospital predisposed 110 beds for COVID-19 patients with different levels of intensity: on the day of the local peak (5th of April), 99 beds were occupied, with a bed occupancy rate (BOR) of 90%. The average COVID-19 BOR over a 45-day period between the 16th of March and the 30th of April was 52.2%.

## Choice of solution

A specific strategy for managing the ICU was necessary, considering the high admission rate reported [2, 3]. Differently from other centres, a dramatic increase of ICU beds was not planned. Rapid and safe discharge of post-critical patients to newly created and equipped step-down units (SDUs) was preferred [4], aiming to improve patient flow and the availability of existing ICU beds.

## Implementation

The Italian COVID-19 crisis began on the 21st of February. During the following two weeks, a new five-bed non-COVID ICU was prepared from the ground up in a physically separated location from the existing ICU: the scope was to maintain a response capacity to neurological, trauma and postoperative patients. Elective surgery was reduced by 80% and limited to non-delayable procedures, while the lockdown caused a 75% reduction of traumas (11 cases versus 44 referred to during the same period in 2019).

The first two COVID-19 patients were admitted to the ICU on 5th of March. The usually existing ICU area (18-bed capacity), equipped with appropriate filter zones, was progressively dedicated to COVID-19 patients. Helped by moderate local epidemiological trends, on the 16th of March, the decision to convert 9 general ward beds into an SDU was taken, instead of increasing ICU capacity. The aim was to create a fluid and manageable unit close to the ICU, acting as a buffer, where patients possibly requiring urgent ICU admission were strictly monitored (coming from other units or directly from the emergency department [ED]), and to rapidly and safely discharge post-critical and not completely weaned patients in case of necessity.

Admission criteria to SDU were as follows: patients at an advanced stage of weaning from mechanical ventilation (MV) and PaO_2_/FiO_2_ > 150, not requiring continuous infusion of sedation or analgesia; patients requiring strict respiratory monitoring and frequent secretions suction, who already started respiratory physiotherapy and a programme for tracheostomy decannulation; patients requiring low or null aminic support; and patients who definitely stopped continuous renal-replacement therapy [5].

SDUs differ from high dependency units (HDUs) or intermediate care units (IMCs) [6]. HDUs and IMCs do not usually admit patients requiring MV [7]. We strategically put the SDU under the control of an experienced intensivist (SDUi). Hospital workforce was relocated to deal with the needs of this new SDU: 5 experienced and well-trained ICU nurses led 15 nurses from other wards or from ORs to cover the unit 24/7. To preserve patients’ and operators’ safety and quality of assistance, patient-per-nurse ratio (2:1), monitoring systems, personal protective equipment (including training to use the devices) and safety procedures were maintained in ICUs. Specific filter zones were created for this new SDUi area. If needed, ventilators were available for non-invasive ventilation (NIV) or MV through tracheostomy (3 cases) and high-flow nasal cannulae (HFNC) oxygen support. The SDU was not considered as an ICU because it was originally a general ward; therefore, it had the disadvantages of tight space, the impossibility to directly observe the patients, 1 intensivist for all 9 patients, and 75% of nurses not used to ICU work.

The ICU and SDUi were formally and administratively independent units, with specifically dedicated (not interchangeable) personnel, just sharing the same informatic system (namely electronic charts and therapies) and the morning briefing: no time was spent on transfer letters, therefore reducing handoffs and communication errors.

A second SDU of 8 beds for COVID-19-positive patients was created on the 23rd of March. It was staffed by pulmonologists (SDUp), with a 2:1 patient-to-nurse ratio. Considering their specific expertise, patients requiring prolonged ventilatory weaning were discharged to the SDUp.

Importantly, while the pandemic was raging nearby and wearing out hospital capacities, local administrators were able to come to the aid of overloaded hospitals, by receiving ICU patients from other centres (25 in total) in a moment of scarce healthcare resources, as well as sending intensivists to support their local teams (frequently infected and thus numerically reduced by COVID-19).

## Evaluation

From general wards or ED, 44 COVID-19 patients were directly admitted to ICU and 8 to SDUi. They were 42 men and 10 women, aged 63.2 ± 10.1 years (mean ± standard deviation [SD]). A total of 14 patients were transferred from the ICU to SDUi (31.8% of 44). A total of 38 ICU patients required MV (86.4%), while only 3 SDUi patients required MV through tracheostomy (13.6% of the 22 patients admitted to SDUi), which was mostly for short periods of recruitment manoeuvres to relieve respiratory fatigue. ICU mortality was 31.8% (14/44), while nobody died in the SDU. Only one readmission to the ICU was recorded, which was due to a bacterial septic shock. Median ICU length-of-stay (LOS) was 11.5 days (interquartile range, 14 days). SDUp received five post-critical patients from the ICU, while their admissions from other units are not available to us.

**Figure 1:**
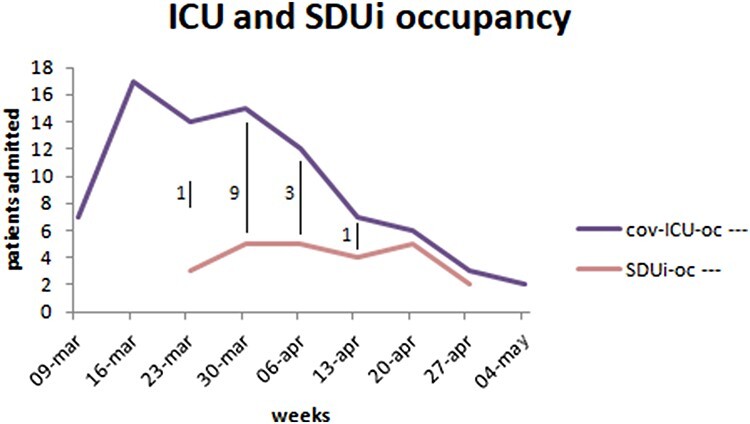
ICU and SDUi occupancy on a weekly basis. On 6 days, between the 26th of March and the 3rd of April, the original 18-bed ICU capacity was exceeded. It is likely that the dramatically increasing number of admitted patients (as visible in the graph up to the 23rd of March) would have required supernumerary ICU beds, with a consequent increase of workload and waiting times for admissions, but the creation of SDUi relieved the situation. cov-ICU-oc: number of occupied beds in the Covid-19 ICU; SDUi-oc: number of occupied beds in the SDUi; vertical black bars report the flow of patients transferred from ICU to SDUi during the whole week (total 14).

A total of 27 beds were dedicated to COVID patients in the ICU and SDUi (18 and 9, respectively). At peak, patients occupied 20 units for 3 days and 19 units for other 3 days, which is higher than the 18 beds available initially, with no system distress. From 30th March to 8th April, 1 to 3 patients were transferred daily from the ICU to SDUi (Figure 1). At its peak, three patients were also admitted from the ED directly to the SDUi in a single day in early April. Despite being set up in just 7 days, SDUi provided supernumerary capacity without system distress at a lower cost and level of intensity, allowing no waiting time for any critical patients requiring ICU admission and higher standards of monitoring and care for worsening patients coming from general wards.

This report has some limitations. One is the description of the strategy applied to our specific situation. There was no ‘control group’ to quantify the benefits; thus, there no statistical analyses were applicable. This approach may be only applicable to places with similar epidemiological trends and institutional dimensions.

## Lessons learned

The COVID-19 pandemic is comparable to a mass casualty incident (MCI) scenario [8, 9], lasting for months with an unpredictable intensity. Our mission is to find out logistical solutions that meet patients’ needs, and secure hospital and primary care resources and local epidemiology and clinical competencies, to maintain a dynamically changing hospital environment. This is essential to reduce the loss of efficiency of our institutions.

We have presented the effects of SDU creation as a possible solution, taking lessons learned from MCIs, to balance the ratio between inflow and outflow of ICU patients. SDUs played an important role in ICU resistance, allowing supernumerary admissions without compromising the safety and quality of care.
